# A complex of measures effectiveness in reducing mother-to-child HIV transmission

**DOI:** 10.1186/1758-2652-13-S4-P162

**Published:** 2010-11-08

**Authors:** SV Minaeva, GF Moshkovich

**Affiliations:** 1 Regional Center for AIDS Control, 20E Minin St., Nizhny Novgorod, Russian Federation; 2 Nizhny Novgorod Regional Center for AIDS Control, Nizhny Novgorod, Russian Federation

## Background

At the early stage of the HIV epidemic in Russia pregnant women who planned having a baby underwent a single HIV testing. Following the sharp increase of the HIV+ number in 2001-2002 (including women) with the aim of reducing mother-to-child HIV transmission risk the Ministry of Health and Social Development recommended providing 2-fold HIV testing for pregnant women during the early stage of pregnancy and before the delivery.

## Purpose of the study

To evaluate the influence of 2-fold HIV testing in pregnant women on HIV- detection, prophylactic interventions (including 3-step prevention), and the risk of HIV transmission to a newborn.

## Methods

We studied HIV incidence and HIV detection in pregnant women and HIV perinatal transmission preventive interventions data, new cases of HIV+ children born in the region in the period of 2004-2009. The years of 2000, 2004 and 2009 have been taken as the key points. Standard statistical methods were used.

## Results

HIV was detected in 5.1 per 100,000 tested pregnant women in 2000, and after the Health Ministry Recommendation was approved HIV was detected in 77.1 per 100,000 tested pregnant women in 2004 and in 61.6 per 100,000 tested pregnant women in 2009. Maximum early detection of HIV infection in pregnant women enabled not only provide mother-to-child HIV transmission prevention but increase the rate of mother-child pairs who received the 3-step prevention (HAART during pregnancy, during delivery, to newborns): in 75% of pregnant women (including 50% 3-stage scheme) in 2000, in 91% (82.1%) in 2004 and in 97.6% (85.8) in 2009. The rate of children perinatally infected reduced and comprised 12.5% in 2000, 10.4% in 2004 and 6.3% in 2009. These measures have led to a great reducing the rate of children infected perinatally: 12,5% in 2000, 10,4% in 2004, and 6,3% in 2009.

## Conclusions

Introducing of the 2-fold HIV testing in pregnant women enabled to 15.1 times increase in HIV detection in pregnant women while there was a 2 time increase in HIV incidence among the whole population as well as the rate of women among all infected (p<0.05). Maximum early preventive interventions extended the 3-step mother-to-child HIV transmission prevention and reduced the risk of newborns infecting.

